# An evolutionary decomposition-based multi-objective feature selection for multi-label classification

**DOI:** 10.7717/peerj-cs.261

**Published:** 2020-03-02

**Authors:** Azam Asilian Bidgoli, Hossein Ebrahimpour-Komleh, Shahryar Rahnamayan

**Affiliations:** 1 Department of Electrical and Computer Engineering, University of Kashan, Kashan, Iran; 2 Nature Inspired Computational Intelligence (NICI) Lab, Department of Electrical, Computer, and Software Engineering, Ontario Tech University, Oshawa, ON, Canada

**Keywords:** Feature selection, Multi-label classification, Multi-objective optimization, Decomposition-based algorithm, Evolutionary algorithm

## Abstract

Data classification is a fundamental task in data mining. Within this field, the classification of multi-labeled data has been seriously considered in recent years. In such problems, each data entity can simultaneously belong to several categories. Multi-label classification is important because of many recent real-world applications in which each entity has more than one label. To improve the performance of multi-label classification, feature selection plays an important role. It involves identifying and removing irrelevant and redundant features that unnecessarily increase the dimensions of the search space for the classification problems. However, classification may fail with an extreme decrease in the number of relevant features. Thus, minimizing the number of features and maximizing the classification accuracy are two desirable but conflicting objectives in multi-label feature selection. In this article, we introduce a multi-objective optimization algorithm customized for selecting the features of multi-label data. The proposed algorithm is an enhanced variant of a decomposition-based multi-objective optimization approach, in which the multi-label feature selection problem is divided into single-objective subproblems that can be simultaneously solved using an evolutionary algorithm. This approach leads to accelerating the optimization process and finding more diverse feature subsets. The proposed method benefits from a local search operator to find better solutions for each subproblem. We also define a pool of genetic operators to generate new feature subsets based on old generation. To evaluate the performance of the proposed algorithm, we compare it with two other multi-objective feature selection approaches on eight real-world benchmark datasets that are commonly used for multi-label classification. The reported results of multi-objective method evaluation measures, such as hypervolume indicator and set coverage, illustrate an improvement in the results obtained by the proposed method. Moreover, the proposed method achieved better results in terms of classification accuracy with fewer features compared with state-of-the-art methods.

## Introduction

In traditional classification approaches, each sample in a dataset belongs to one class. However, in recent years, to adapt to real-world problems, researchers have studied multi-label learning (Zhang & Zhou, 2014). In such problems, each sample in a dataset can simultaneously belong to several classes. Therefore, a set of labels is defined for each data entity. Because this is supervised learning, the objective of the classification is to create a model by using the training data to predict the unseen data labels. In real-world applications, it is less common for each entity to have exactly one label; for this reason, this is an important direction for research. In multi-label text classification, each text sample can simultaneously belong to different classes (such as “politics” and “sports”) (Ueda & Saito, 2003). Another example is digital image classification: an image sample may contain a mountain, lake, and tree; hence, the image is included in each of the classes (Boutell et al., 2004). In the functional classification of genes, every gene is also a member of different functional classes (such as “metabolism” and “protein synthesis”) (Li, Miao & Pedrycz, 2017).

The accuracy of a classification task strongly depends on the selected features that provide the most relevant knowledge about the data to construct a reliable model. Feature selection is a data mining preprocessing task that removes irrelevant and redundant features. It reduces computational complexity in the learning process and improves the classifier’s performance (Zhang, Gong & Rong, 2016). In multi-label datasets, each sample is related to more than one label, and the corresponding labels are not necessarily independent of each other; hence, feature selection in such a dataset is more complicated than in single-label classification (Zhang et al., 2017). Several researchers have reported that classification performance can be improved using a proper feature selection strategy in multi-label data (Madjarov et al., 2012; Lee & Kim, 2015; Dembczynski et al., 2012). The feature selection methods for both multi-label and single-label datasets can be divided into three categories: wrapper, filter, and embedded methods (Pereira et al., 2016). The wrapper methods select the features based on the resulting classification performance; hence, the learning task is a part of the feature selection process. Additionally, wrapper methods have been used for multi-label data feature selection (Dendamrongvit, Vateekul & Kubat, 2011; Wandekokem, Varejão & Rauber, 2010). In filter methods, the best set of features is selected using the statistical characteristics of data (e.g., the correlation among features and classes). Many filter-based feature selection methods have been proposed for multi-label data (SpolaôR et al., 2013a, 2013b; Reyes, Morell & Ventura, 2015; Lin et al., 2016; Li, Miao & Pedrycz, 2017). The embedded methods select best subset of features as a integrated part of the learning algorithm. One of the well-known embedded methods is Decision Tree algorithm (Safavian & Landgrebe, 1991). This classifier constructs a tree structure model which selects best feature at each node in term of a discriminancy criterion. To obtain the best subset of features out of *d* features, we need to evaluate 2^*d*^ possible subsets. Consequently, selecting the best subset out of all possible subsets is extremely time-consuming; therefore, it is not practical to employ a brute force approach. In fact, feature selection is an NP-hard problem (Chandrashekar & Sahin, 2014; Blum & Langley, 1997). Therefore, the use of meta-heuristic search strategies, such as evolutionary algorithms, can be beneficial in this regard (Ibrahim et al., 2019b, El Aziz & Hassanien, 2018; Elaziz et al., 2017; Mousavirad & Ebrahimpour-Komleh, 2014). Evolutionary algorithms have attracted significant attention because they are more robust in avoiding local optima, compared with traditional optimization methods (Ibrahim et al., 2019a; Elaziz et al., 2020). Various evolutionary algorithms have been used for multi-label feature selection (Zhang, Pena & Robles, 2009; Lee & Kim, 2015; Reyes, Morell & Ventura, 2014; Shao et al., 2013).

Some studies in feature selection have considered only classification accuracy for their optimization algorithm, whereas several other objectives can be simultaneously optimized using multi-objective optimization algorithms. Although feature selection can enhance the accuracy of the classification task and decrease the computational complexity, an extreme reduction of relevant features will degrade the accuracy. On the other hand, increasing the number of appropriate features gives more relevant knowledge of data to construct an accurate model. Accordingly, a massive number of features increases the computational complexity of a classification task because of the complexity of its search space. Therefore, the main goal of multi-objective feature selection has two conflicting objectives, that is, to minimize the number of features while maintaining an acceptable classification accuracy (Dembczynski et al., 2012; Xue, Zhang & Browne, 2013).

To the best of our knowledge, a few articles have used multi-objective optimization methods for feature selection of multi-label data. Yin, Tao & Xu (2015) attempted to find the best subset of features by using the nondominated sorting genetic algorithm II (NSGA-II) (Deb et al., 2000). In another study, feature selection in multi-label datasets used a differential evolution algorithm (Zhang, Gong & Rong, 2016). Zhang et al. (2017) presented a particle swarm optimization (PSO)-based multi-objective optimization algorithm and achieved a better accuracy compared with the previous methods. Lee, Seo & Kim (2018) proposed an evolutionary multi-label feature selection that used dependencies between the features and labels to select more relevant features. Their method selects features that have a higher level of correlation with the labels and have not been selected using genetic operators during the optimization process. In another study , the most salient features were selected by mapping the features to a multi-dimensional space based on the correlation between features and each label (Kashef & Nezamabadi-pour, 2019). However, the authors have only used the Pareto-dominance concept inspired by multi-objective optimization. In other words, they do not search the features’ space using a multi-objective optimization algorithm.

Evolutionary-based multi-objective optimization algorithms can be divided into three categories: dominance-based, decomposition-based, and indicator-based methods (Trivedi et al., 2017). The dominance-based methods attempt to find the solutions that optimize the objective functions by using a concept called dominance, which will be defined in the next section. All the above-mentioned studies on multi-label feature selection belong to this category of multi-objective optimization algorithms. The indicator-based methods evaluate the fitness of each solution by assessing an indicator (such as hypervolume) to improve the convergence and diversity criteria simultaneously. On the contrary, the decomposition-based methods decompose the whole search space into smaller subproblems and solve all of them simultaneously. Therefore, the convergence rate of the algorithm is significantly improved, which enhances the diversity of the obtained solutions (Zhang & Li, 2007). An advantage of decomposition-based methods is their potential for scalability to multi-objective optimization problems (Zhang & Li, 2007).

Research on feature selection for multi-label data has started recently; therefore, few studies have been conducted in this area, especially for multi-objective problems. The most important aim of this paper is to address this problem for multi-objective optimization.

In this article, we propose a decomposition-based method for multi-label feature selection. The objective functions used in this paper include Hamming loss and the number of features. The main contributions of the paper can be summarized as follows: (1) we address the problem of multi-objective feature selection by solving several single-label subproblems, that is, for the first time, decomposition-based evolutionary multi-objective optimization has been used for multi-label classification; (2) we apply a local search strategy to increase the exploitation power of the proposed method; (3) we propose a hybrid crossover scheme that switches among crossover operators with a predefined probability. Because some of the benchmark datasets have more than 1,000 features, we used decomposition-based algorithms, which are beneficial for large-scale problems.

To validate the results, we compared the proposed method of multi-label feature selection with state-of-the-art methods. Furthermore, to validate the performance of the proposed algorithm, we conducted an extensive set of experiments on real-world multi-label datasets. The results show a significant improvement compared with the other methods in terms of multi-objective evaluation measures, such as hypervolume indicator and set-coverage criterion.

This article is organized as follows. “Background Review” describes related work on multi-label classification, multi-objective optimization, and the existing methods for multi-objective multi-label feature selection. The proposed algorithm is explained in “Proposed Method”. The experiments are presented in “Experimental Design”. “Results and Discussion” describes and discusses the results. Finally, “Concluding Remarks” concludes the article.

### Background review

In the following subsections, we briefly review related concepts. We start with a brief explanation of multi-label classification to clarify the importance of this research problem. Next, we explain multi-objective optimization and the corresponding challenges. Finally, we examine existing multi-label feature selection methods that have been proposed for multi-objective optimization algorithms.

### Multi-label classification

If a dataset *X* contains *d*-dimensional samples and *Y* represents the set of the *q* possible labels in a multi-label problem, the objective of multi-label classification is to create a model in the form of $h:X \to {2^Y}$ from *m* training examples, *D* = (*X*_*i*_, *Y*_*i*_|1 *≤ i ≤ m*). For each multi-label sample (*X*_*i*_, *Y*_*i*_), *X*_*i*_ includes a *d*-dimensional feature vector and *Y*_*i*_ includes a set of labels associated with *X*_*i*_. For unseen data *X*_*i*_, the multi-label classifier predicts *h*(*X*) as the set of labels. Multi-label learning algorithms can be divided into two main categories: problem transformation and algorithm adaptation methods (Zhang & Zhou, 2014). In the problem transformation methods, the multi-label classification problem is converted into a single-label problem to classify data using existing single-label classifiers. The basic idea of the algorithm adaptation methods is to adapt single-label classifiers to deal with multi-label data. Multi-label *K*-nearest neighbor (ML-KNN) (Zhang & Zhou, 2007) is one of the most well-known adaptive methods, and it was used in this study to evaluate feature subsets. In the single-label version of this algorithm, to predict the class label of the sample, the algorithm calculates the distance between the query sample and the other samples in dataset. *K* neighbors (smallest distances) of the sample should be picked. The algorithm gets the labels of the selected *K* entries. Then it returns the mode of the *K* labels as the class of query sample. Despite its simplicity, this classifier is commonly used on various applications. In its multi-label version ML-KNN, as in the single-label version, the sample would be labeled by classes in which the distribution of neighbors is higher. In this direction, decision making is performed for every class as follows:

(1) $$Y = \{ {y_i}|P({H_j}|{C_j})/P({\rm \sim }{H_j}|{C_j}) \gt 1,1 \le j \le q\}$$

Sample *x* belongs to class *j* if the posterior probability *P*(*H*_*j*_|*C*_*j*_) that *x* belongs to class *j*, providing that *x* has exactly *C*_*j*_ neighbors with label *y*_*j*_, is bigger than *P*(∼*H*_*j*_|*C*_*j*_). To obtain the value of posterior probability, Bayes’ theorem mentioned in Eq. (2) has been applied. The ratio of two mentioned posterior probabilities determines belonging of the sample to class *j*. According to this equation, the posterior probability is dependent on the values of prior probabilities (*P*(*H*_*j*_) and *P*(∼*H*_*j*_)) and likelihood functions (*P*(*C*_*j*_|*H*_*j*_) and *P*(*C*_*j*_|∼*H*_*j*_)).

(2) $$\displaystyle{{P({H_j}|{C_j})} \over {P({\rm \sim }{H_j}|{C_j})}} = \displaystyle{{P({H_j})\cdot P({C_j}|{H_j})} \over {P({\rm \sim }{H_j})\cdot P({C_j}|{\rm \sim }{H_j})}}$$

To calculate the *P*(*H*_*j*_), we obtain the ratio of the samples that have label *y*_*j*_ to the total samples. The value of *P*(*C*_*j*_|*H*_*j*_)) is also calculated using Eq. (3), where *k*_*j*_(*r*) is the number of samples in the training set that have label *y*_*j*_ and have exactly *r* neighbors with label *y*_*j*_. Based on this definition, *k*_*j*_(*C*_*j*_) is the number of samples that belong to class *j* and have *r* neighbors in this class.

(3) $$P({C_j}|{H_j}) = \displaystyle{{{k_j}({C_j})} \over {\displaystyle\sum\nolimits_{r = 0}^k {{k_j}(r)} }}\quad (1 \le j \le q,\ 0 \le {C_j} \le k)$$

Because of the simplicity and popularity of ML-KNN, we used this classifier to evaluate the quality of selected features in our proposed method. Moreover, we use the same classifier to compare several algorithms.

### Multi-objective optimization

Most real-world optimization problems involve multiple conflicting objectives (Konak, Coit & Smith, 2006). Hence, multi-objective optimization problems have various practical applications. The use of evolutionary algorithms has been motivating for solving such problems. Because of the population-based nature of these algorithms, we obtain a set of solutions on every run. In a multi-objective optimization problem, the definition of optimality is not as simple as in single-objective optimization. When the optimal solution of an objective function conflicts with an optimal solution of another objective function, the problem becomes challenging. Therefore, to solve such problems, it is necessary to find a trade-off between objective functions. The obtained solutions of multi-objective algorithms are called nondominated solutions or Pareto-optimal solutions. Theoretically, if a multi-objective optimization problem is a minimization problem, it is formulated as follows (Mirjalili et al., 2016).

(4) $$\matrix{\hfill  {{\rm Min}\quad F({\boldsymbol{x}}) = [{f_1}({\boldsymbol{x}}),{f_2}({\boldsymbol{x}}),\ldots ,{f_M}({\boldsymbol{x}})]} \hfill \cr  {{\rm s.t.}\quad {L_i} \le {\boldsymbol{x}_i} \le {U_i},\quad i = 1,2,\ldots ,d} \hfill \cr }$$

Subject to the following equality and inequality constraints:

(5) $$\matrix{ {{g_i}({\boldsymbol{x}}) \le 0\quad j = 1,2,\ldots,J} \hfill \cr {{h_k}({\boldsymbol{x}}) = 0\quad k = 1,2,\ldots,K} \hfill \cr }$$

where *M* is the number of objectives, and *d* is the number of decision variables (dimension) of solution ***x***, so that ***x***_*i*_ should be in interval [*L*_*i*_,*U*_*i*_] (i.e., box-constraint). Finally, *f*_*i*_ is the objective function that should be minimized. To compare two candidate solutions in multi-objective problems, we can use the concept of Pareto dominance. Mathematically, the Pareto dominance is defined as follows. If ***x*** = (*x*_1_, *x*_2_,…, *x*_*d*_) and $\acute{\boldsymbol x} = (\acute{x}_1, \acute{x}_2,\ldots , \acute{x}_d)$ are two vectors in the search space, ***x*** dominates ${\acute{x}}({\boldsymbol{x}}\succ \acute{\boldsymbol x})$ if and only if

(6) $$\matrix{ \hfill  {\forall i \in \{ 1,2,\ldots ,M\} ,{f_i}({\boldsymbol x}) \le {f_i}({\acute{\boldsymbol {x}}}) \wedge } \hfill \cr \hfill  {\exists j \in \{ 1,2,\ldots ,M\} :{f_j}({\boldsymbol x}) \lt {f_j}({\acute{\boldsymbol {x}}})} \hfill \cr }$$

This means that solution ***x*** dominates solution $\acute{\boldsymbol{{x}}}$ (is better) if and only if the objective values of ***x*** are better than or equal to all objective values of $\acute{\boldsymbol{{x}}}$ (is not worse than $\acute{\boldsymbol{{x}}}$ in any of the values of the objective functions) and it has a better value than ***x*** in at least one of the objective functions. If the solution ***x*** is better than $\acute{\boldsymbol{{x}}}$ in all objectives, we call strong dominance but in the case that they have at least one equal objective, the weak dominance happens. All nondominated solutions construct a Pareto front.

Crowding distance (Deb et al., 2000) is another measure to compute the distribution of candidate solutions in the objective space. It is calculated using the sum of distances between each solution and its neighbors. It is computed using Eq. (7).

(7) $$C{D_i} = \sum\limits_{j = 1}^M |{f_j}(i + 1) - {f_j}(i - 1)|,$$

where *f*_*j*_(*i* + 1) and *f*_*j*_(*i* − 1) indicate the *j*th objective value of the previous and next neighbors of solution *i*. Larger distance indicates a non-crowded space. Hence, a selection of solutions from this region creates a better distribution. In fact, it represents the distribution of the members surrounding each solution. Decomposition-based methods are a category of multi-objective optimization algorithms that decompose the approximation of PF into a number of single-objective optimization subproblems. All subproblems can be solved by using other classical optimization or evolutionary methods simultaneously. A strategy is required to convert a multi-objective optimization problem to single-objective one. During optimization, the trade-off relation between objectives can be applied by considering information mainly from subproblem neighboring. The neighborhood concept is defined by producing a set of weight vectors in objective space. If the subproblems are solved by evolutionary algorithms, neighbors communicate with each other to reproduce the new candidate solutions and update the existing individuals in the population. The steps of a decomposition-based method has been explained in “Proposed Method” section.

### Tchebycheff method

As stated before, multi-objective optimization problems can be solved by different methods. Traditional multi-objective optimization methods seek a way to convert the multi-objective problem into a single-objective problem. One of these methods is the Tchebycheff method (Jaszkiewicz, 2002), which was used in this study to solve multi-objective subproblems. The Tchebycheff method looks for the optimal solutions that have the minimum distance from a reference point. The single-objective optimization problem is defined as Eq. (8).

(8) $$\matrix{ \hfill  {{\rm Minimize}\quad {g^{te}}(x|{{\rm \lambda} _o},{z^*}) = {\rm max}\{ {{\rm \lambda} _i}|{f_i}(x) - z_i^*|\} } \hfill \cr {{\rm subject\,\,to}\quad x \in S,} \hfill \cr }$$

where z* = (*z*_1*_,…,*z*_m*_)^*T*^ is a reference point used to evaluate the quality of the obtained solutions, *m* is the number of objective functions, and *S* is the search space. According to this equation, the distances between the objective function values of each solution *x* and reference point z* are calculated. The single-objective optimization problem is regarded as minimizing the maximum of these distances. A uniform weight vector λ = (λ_1_, λ_1_,…, λ_*m*_) is defined for each solution such that $\sum\nolimits_i^m {\lambda _i} = 1$. Therefore, weight *λ*_*i*_ is assigned to the objective function *f*_*i*_. To obtain each optimal solution of the minimization problem defined in Eq. (8), we need to find an appropriate weight vector. The obtained optimal solution would be one of the Pareto optimal solutions. As a result, the traditional methods are time-consuming because of continuous changes in the weights required to obtain the best solutions. Therefore, we consider a set of distributed weight vectors in the decomposition-based evolutionary methods for all the subproblems. Reference point selection is another issue that should be considered in the Tchebycheff method. For a minimization problem, the minimum value obtained for each objective function can be a reference point.

(9) $$z_i^* = {\rm min}{f_i}(x)|x \in S$$

**Figure 1:**
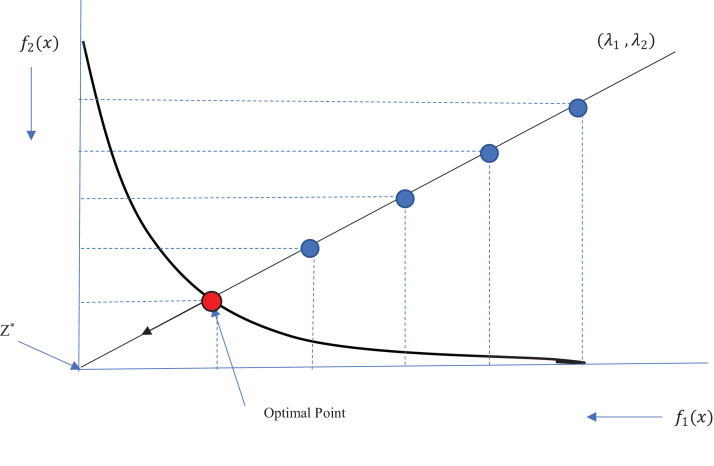
Illustration of Tchebycheff method.

Therefore, the value of the reference point is also updated after each iteration. Figure 1 shows the Tchebycheff method for obtaining an optimal solution on the Pareto front. As an example, we show that the reference point has been placed at the center of the coordinates, where the values of both objective functions are minimal. We show a sample weight vector (λ_1_, λ_2_), and the solutions from each iteration are shown in blue. The solutions converge toward the reference point in the direction of the weight vector until the optimal point on the Pareto front (in red) is obtained. At each iteration, the previous solution is replaced with a new solution if the new one outperforms the previous one.

### Multi-label feature selection using multi-objective evolutionary algorithms

A review of the literature shows little research in the area of multi-label feature selection using multi-objective evolutionary algorithms. Next, we briefly explain the state-of-the-art methods.

#### Multi-label feature selection algorithm based on NSGA-II

Yin, Tao & Xu (2015) have selected the optimal features for multi-label data classification using the NSGA-II algorithm. The Hamming loss and the average precision criteria have been considered as the objective functions. This paper has yielded the Pareto front using the NSGA-II algorithm. NSGA-II uses fast non-dominated sorting to rank feature subsets. The fast non-dominated sorting technique categorizes the population members in different ranks. For each solution *p*, the number of members for which solution *p* dominates and the number of members that dominate solution *p* are specified.

All solutions that have never been dominated (members with a domination count of zero) are added to a set named *F*_1_. Here, *F*_1_ is the first Pareto front that contains the best-qualified members of the current population. In the next step, the members included in *F*_1_ are removed from the population, and the remaining members that have never been dominated construct the second rank *F*_2_. This procedure continues in the same way until all population members are ranked.

At the end of the algorithm, the members of the first front *F*_1_ are presented as the optimal Pareto front. The proposed method was tested on multi-label standard data classified using the ML-KNN classifier. The authors compared the proposed method with several filter-based feature selection methods.

#### PSO-based multi-objective multi-label feature selection

PSO is a well-known population-based evolutionary algorithm. Zhang et al. (2017) presented a multi-objective PSO-based method for feature selection of multi-label data. They considered the number of features and accuracy of classification as conflicting objectives. In the PSO algorithm, population consists of particles that have two properties: position and velocity. The position and the velocity of the *i*-th particle are presented as *P*_*i*_(*t*) = (*p*_*i*,1_, *p*_*i*,2_,…, *p*_*i*,*D*_) and *V*_*i*_(*t*) = (*v*_*i*,1_, *v*_*i*,2_,…, *v*_*i*,*D*_), respectively. The position of the particle is updated based on the previous position and velocity. Moreover, the particle velocity is updated according to Eq. (10) based on two parameters: (1) the best individual position of the particle up to now *Lb*_*i*_(*t*) = (*lb*_*i*,1_, *lb*_*i*,2_,…, *lb*_*i*,*D*_) and (2) the best global position among all particles *Gb*(*t*) = (*gb*_1_, *gb*_2_,…, *gb*_*D*_).

(10) $${{v_{i,j}}(t + 1) = } {w \times {v_{i,j}}(t) + {r_1} \times {c_1} \times (l{b_{i,j}}(t) - {p_{i,j}}(t)) + {r_2} \times {c_2} \times (g{b_j}(t) - {p_{i,j}}(t))}$$

(11) $${p_{i,j}}(t + 1) = {p_{i,j}}(t) + {v_{i,j}}(t + 1)$$

where *t* is the number of iterations; *r*_1_ and *r*_2_ are two random vectors uniformly distributed in the range (0, 1); *c*_1_ and *c*_2_ are two parameters that represent the particle’s confidence in itself and in the swarm, respectively, and *w* determines the effect of previous velocity, called inertia weight. Generating an initial population is the first step of PSO-based multi-label feature selection. Then, an archive of nondominated solutions is provided. Velocities and positions of all particles are updated in each iteration. We also need to update the particle’s best individual position and the best global position. The particle’s best individual position is calculated using the domination concept. In addition, the best global position is selected among the particles’ historical positions by using the crowding distance criterion. An adaptive mutation operator has been used to produce offspring; the number of the mutated elements in a particle is determined using a non-linear function. For this purpose, *K* variables of some particles are randomly selected to be reinitiated. The proposed method has been evaluated on standard benchmark problems using the ML-KNN classifier. The results show significant improvements compared to the previous state-of-the-art methods.

## Proposed Method

Decomposition methods (such as the Tchebycheff method) are traditional methods of multi-objective optimization. They transform the problem of approximation of the Pareto front into a number of scalar optimization problems. As mentioned before, because of the continuous modifications of the objective functions’ weights for obtaining a Pareto solution, these methods may be time-consuming. Some of them are unable to discover all Pareto points in convex problems effectively (Zhang & Li, 2007). An evolutionary algorithm can be used to overcome this problem. Recently, a method based on decomposition and evolutionary algorithms (MOEA/D) was proposed for solving a multi-objective problem (Zhang & Li, 2007). MOEA/D uses evolutionary algorithms for decomposition of the problem space into scalar subproblems and simultaneously solves them. Hence, it increases the speed of finding Pareto-optimal solutions and improves the diversity of the obtained solutions (Zhang, Gong & Rong, 2016). The scalar subproblems are simultaneously solved by receiving information from neighboring subproblems; therefore, the algorithm has less computational complexity compared to the domination-based algorithms. MOEA/D has several advantages over Pareto dominance-based algorithms, such as computational efficiency, scalability to optimization problems with many objectives , and high search ability for combinatorial optimization problems (Jiang et al., 2011).

**Figure 2:**
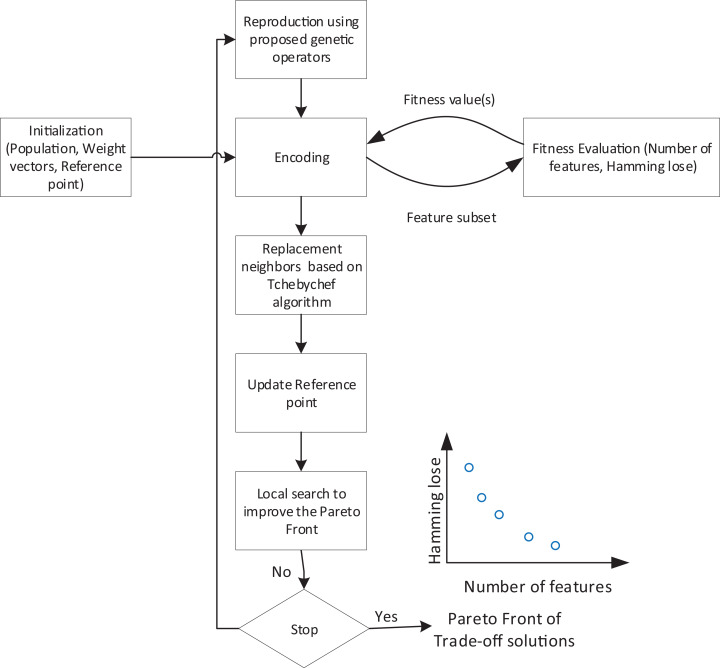
Flowchart of overall structure of the proposed method.

In this article, we propose a decomposition-based multi-objective feature selection for multi-label data classification. This is the first time that a decomposition-based approach has been customized to tackle multi-label classification. Figure 2 shows the overall flowchart of the proposed method. According to the overall structure, the search process needs an encoding strategy to define the search space which is explained in the next subsection. Algorithm, as an iterative process, starts with initialization step. At each iteration, based on the proposed operators, new feature subsets are created and evaluated using objective functions. Using Tchebychef method, the neighbors of generated solutions and reference points will be updated. After applying a local search, a set of non-dominated solutions are obtained as trade-off feature subsets. Algorithm 1 also represents the Pseudo-code of the proposed method which in the following subsections, we describe the details of its main components.

**Algorithm 1.** Pseudo-code for the proposed method.

**input : ***NP*: the number of subproblems, T: the number of neighbors in the decomposition-based optimization algorithm, *K*: the number of neighbors in the multi-label KNN classifier, *R*: the number of iterations
**output** : final feature selection subsets
// Initialization
1 Divide multi-label data into two training and test sets;
2 Produce the weight vectors by uniformly distributed aggregation values;
3 Generate the initial population uniform randomly;
4 Evaluate the objective functions for each candidate solution according to (Eq. 12) using training set;
5 Compute the *T* neighbors for each weight vector using Euclidean distance;
6 Initialize the reference point according to (Eq. 9);
7 Determine the non-dominated solutions in the initial population as an archive (AC);
8 it=0;
// Main algorithm
9 **while** *it* < ***R* do**
10 **for *i***←**1 to** *NP* **do** // For each individual, *x*_*i*_, in the population
// Regeneration
11 Randomly select two candidate solutions from among the neighbors of *x*_1_;
12 Produce two new candidate solutions, *y*_1_, *y*_2_ using the proposed genetic operators;
// Comparison and replacement (Eq. 8)
13 **for** *j*←1 **to** *T* **do** // For each neighbor
14 **if** *g*^*te*^(*y*_1_|*W* ^*j*^,Z) ≤ *g*^*te*^(*x*_1_|*W* ^*j*^ ,*Z*) **then**
15 *x*_*j*_ = *y*_1_
16 **end**
// Update the reference point (Eq. 9)
17 **if** *f*_1_(*y*_1_) < *z*_1_ **then**
18 *z*_1_ = *f*_1_(*y*_1_)
19 **end**
20 **if** *f*_2_(*y*_1_) < *z*_2_ **then**
21 *z*_2_ = *f*_2_(*y*_1_)
22 **end**
23 **if** *g*^*te*^(*y*_2_|*W*^*j*^ ,*Z*) ≤ *g*^*te*^(*x*_1_|*W* ^*j*^ ,*Z*) **then**
24 *x*_*j*_ = *y*_2_
25 **end**
// Update the reference point (Eq. 9)
26 **if** *f*_1_(*y*_2_) < *z*_1_ **then**
27 *z*_1_ = *f*_1_(*y*_2_)
28 **end**
29 **if** *f*_2_(*y*_2_) < *z*_2_ **then**
30 *z*_2_ = *f*_2_(*y*_2_)
31 **end**
32 **end**
33 **end**
// Local Search and obtaining final Pareto
34 Separate non-dominated solutions from the updated population (NS);
35 Separate non-dominated solutions from AC and NS (EP);
36 Select a solution with the maximum crowding distance (*X*_*cr*_) and two random solutions *X*_*n1*_, *X*_*n2*_ from EP;
37 Produce a new solution by using (Eq. 13);
38 Select the non-dominated solutions as the final Pareto set from EP and $\acute{X}$;
39 Update the archive;
40 *it* = *it* +1;
41 **end**
42 Obtain the hamming loss for test data with the selected features of solutions in the final Pareto front.

### Representation of individuals

**Figure 3:**
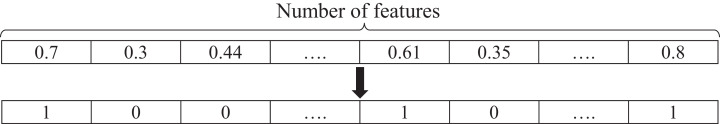
An instance of a feature selection representation.

Each member of the population indicates a candidate solution in the search space. In this paper, the representation of individuals for feature selection is a string with a length equal to the number of features. A cell of the vector is randomly filled with a real value between 0 and 1. This representation is used in problems that need a continuous representation of the solutions (Xue, Zhang & Browne, 2013). The use of real values is due to the use of continuous genetic operators. A cell with a value greater than 0.5 indicates the selection of the feature, and a value less than 0.5 indicates that a feature is not selected. If the length of the feature vector is *D*, the *i*-th population member is defined as *c*_*i*_(*t*) = (*c*_*i*,1_, *c*_*i*,2_,…, *c*_*i*,*d*_). The feature subsets use the following notation: when a feature is selected, the corresponding cell value changes to 1; otherwise, it becomes zero. Hence, the string is converted to a binary vector, where 0 indicates the rejection of the feature, and 1 indicates the selection of the feature. Therefore, the number of selected features is equivalent to the count of “1” in the vector. An instance of a feature vector is shown in Fig. 3.

### Objective functions

To acquire the best solutions in feature selection, we consider two objective functions: the number of selected features and the Hamming loss. As mentioned before, the goal of feature selection is to remove irrelevant and redundant features and, therefore, to reduce the complexity of the search space in the classification task or any other feature-based process. The ratio of the features selected for each solution to all the features (a value between 0 and 1) is our first objective function. The second objective function evaluates the learning accuracy of multi-label data. The Hamming loss is one of the most well-known measures for computing the classification error for multi-label data; it has been used in several papers on multi-label wrapper feature selection (Zhang et al., 2017; Yin, Tao & Xu, 2015; Jungjit & Freitas, 2015). The Hamming loss evaluates the fraction of misclassified instance-label pairs, that is, a relevant label is missed, or an irrelevant one is predicted (Zhang & Zhou, 2014). The Hamming loss is defined as follows:

(12) $$hloss(h) = \displaystyle{1 \over p}\sum\limits_{i = 1}^n \displaystyle{1 \over q}|h({x_i})\Delta {Y_i}|,$$

where *q* is the number of labels, and *p* is the total number of multi-label samples. If *x*_*i*_ shows the *i*-th sample, *h*(*x*_*i*_) represents the labels predicted by model *h*. Moreover, *Y*_*i*_ are the actual labels of the *i*-th sample. Δ is the difference between the vectors of the predicted and actual labels. The Hamming loss error is our second objective function for feature selection. Hence, multi-objective optimization can be applied to minimize this objective as well. According to the definitions of the two objective functions, the proposed method attempts to find feature sets with a minimum number of features and a minimum classification error.

### The proposed genetic operators

**Figure 4:**
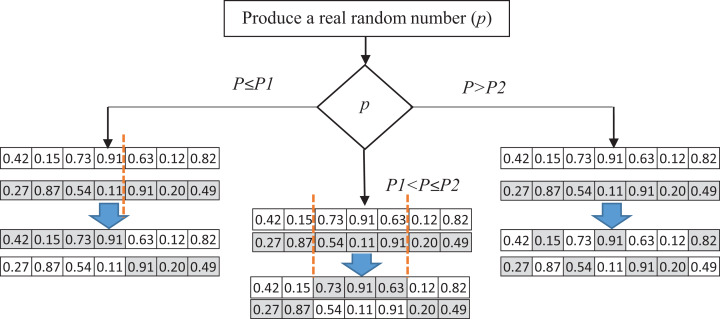
The process of selecting crossover operators.

In this paper, we introduce a pool of crossover operators to obtain the benefits of various operators to produce better solutions. Three genetic operators—single point, double point, and uniform crossover—are used to produce a new generation of candidate solutions. In each iteration, one of these crossover operators is selected. A random number *P* between 0 and 1 is generated as the selection probability of one of the operators. The ranges of the selection are specified using *P*_1_ and *P*_2_, which can be determined in the experiments. If the generated number is less than *P*_1_, the single-point crossover is applied to the parent solutions. In the single-point crossover, a random point is selected, and the tails of its two parents are swapped to generate new offspring. The double-point crossover is selected if *P* is between *P*_1_ and *P*_2_. The double-point crossover is a generalization of the single-point crossover wherein alternating segments are swapped to generate new offspring. A probability greater than *P*_2_ causes the selection of the uniform crossover to produce offspring. It performs the swapping of the parents by choosing a uniform random real number (between 0 and 1). The random real number decides whether the first child selects the *i*th gene from the first or the second parent. For each variable, a uniform random number is generated. Based on the value of this number, the child’s variable is selected from one of the parents. If the random number is more than 0.5, the first parent’s variable would be selected, and vice versa. Figure 4 shows the process of selecting crossover operators.

A uniform mutation is applied for a newly produced individual to guarantee the diversity property. A random number of features is selected from the generated subset. Then, the values of the variables related to the corresponding features will be replaced with a new random uniform number between 0 and 1.

### Local search

The domination concept is used to separate the best candidate solutions at the end of each iteration. All dominated solutions are omitted from the population. To improve the obtained Pareto front in the decomposition-based algorithm, a local search (Zhang et al., 2017) is applied to produce a candidate solution in the search space with a large crowding distance. We estimate the density of solutions surrounding each solution; hence, producing a new solution in the area with less density is desirable. For this purpose, at the end of each iteration, the final Pareto front is saved in the archive (AC). A solution with the maximum crowding distance (*X*_*cr*_) is selected among non-dominated solutions of the new Pareto front obtained from the current generation and the solutions in the archive (from the previous generations). A new solution is produced by using *X*_*cr*_ and two random solutions, *X*_*n*1_, *X*_*n*2_, based on the following equation:

(13) $${\acute{X}_i} = {X_{cr}} + F \cdot ({X_{n1}} - {X_{n2}})$$

Parameter *F* is a scale factor that amplifies the difference between the two vectors. The final Pareto front will include nondominated solutions among the newly produced solutions in the local search and AC; this local search is inspired by the differential evolution (DE) algorithm (Price, Storn & Lampinen, 2006).

### Overall structure of the proposed method

**Figure 5:**
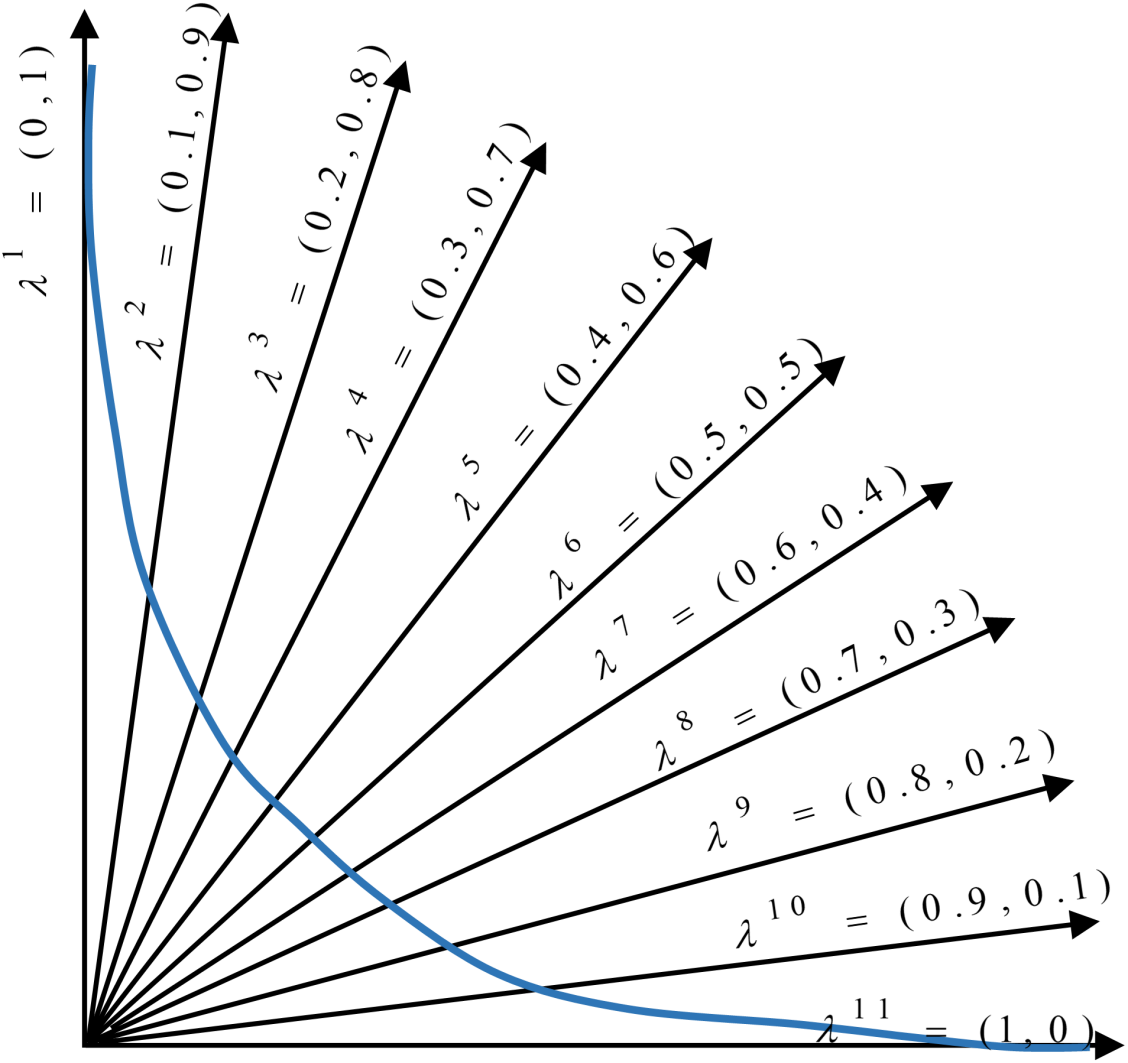
Distribution of weight vectors in a minimization problem.

In the proposed method, the multi-objective problem is divided into scalar subproblems, and the best solution is simultaneously searched in each subproblem using an evolutionary algorithm. An appropriate Pareto front will be achieved by solving each subproblem. Figure 5 illustrates the general idea of a minimization problem with two objectives and 11 subproblems. As seen, a composition function *g* converts two objectives (*f*_1_ and *f*_2_) into one scalar objective. In addition, there exists a vector (with its dimensions equal to the number of objectives) that weights each objective in the composite function. According to the figure, the search space is divided into 11 sections using uniformly distributed aggregation weight vectors. Each section has a different weight vector, and each weight vector determines a search direction. The weight vectors are generated using Das and Dennis’ method (Das & Dennis, 1998). In this approach for constructing the weight vectors, we apply the uniform distribution scheme. Let *N* be the number of subproblems, and λ^1^, λ^2^,…, λ^*N*^ be the weight vectors, where each weight has a value from {0, 1/*N*, 2/*N*,…,1}. The sum of the individual weights of every subproblem should be equal to one. In the proposed method, ${\rm \lambda} _1^1$ is the weight of the first objective (number of features) and ${\rm \lambda} _2^1$ is the weight of the second objective (the Hamming loss) in the first subproblem.

Each subproblem includes the following parts:

- An individual *X*_*i*_
- The objective functions (i.e., the number of features and hamming loss, are considered)
- A weight vector λ^*i*^
- The neighborhood of subproblem *i*
- Composite objective function *g*^*te*^ based on Eq. (8).

The number of subproblems is usually considered equal to the population size. In each generation, we form a population of the best solutions found for each subproblem. The neighborhood relations among subproblems are defined based on the distance between the weight vectors. During the search, we produce new solutions for each subproblem by the cooperation of neighborhood members and by using the proposed genetic operators (selection, crossover, and mutation). The new solutions compete with the neighbors of the old solutions; specifically, we compare weighted combinations of two objective functions for different solutions. The function weights indicate the moving direction of the population for finding an optimal solution. Subsequently, the steps of the proposed algorithm are explained in detail.

Step 1. Initialization: In this step, subproblems should be initialized. The proposed method starts by generating an initial population and weight vectors. The structure of each solution in the population is described in “Representation of Individuals”. There is a weight vector corresponding to every subproblem that is used to convert the multi-objective problem into a single-objective problem by using the Tchebycheff method. As a result, the number of subproblems, the number of candidate solutions in the population, and the number of weight vectors are the same. The length of the weight vector would be equal to the number of objective functions. Different uniformly distributed aggregation weight vectors distribute the candidate solutions in the whole search space; hence, a Pareto front with appropriate diversity is achieved. In the subproblems where larger weight values are assigned to the first objective function, the number of features becomes more important than classification performance. Therefore, the composite function forces the subproblem to find solutions with fewer features. In addition, we use the neighborhood concept to produce new solutions and improve the available solutions in the decomposition-based methods. Therefore, for each subproblem, the closest weight vectors are calculated based on the Euclidean distance from their neighbors. The values of the objective functions for all solutions in the initial population need to be calculated. Therefore, the ML-KNN classifier is applied to the training data with the feature subset of each candidate solution. Furthermore, at the beginning of the algorithm, the reference point should be initialized to evaluate the candidate solutions using the Tchebycheff method. In the proposed method, the minimum values of the objective functions among all the obtained candidate solutions are regarded as the reference point. Point *z* = (*z*_1_, *z*_2_) is considered as the reference point, where *z*_1_ is the minimum number of the selected features among the obtained solutions, and *z*_2_ is the minimum value of the classification error. This step is shown in lines 1–8 of Algorithm 1.

Step 2. Regeneration: Producing new candidate solutions (offspring) is one of the main steps of evolutionary algorithms. In the proposed method, the genetic operators (explained in the previous section) were used to produce new offspring. At every iteration, for each available subproblem, a new candidate solution should be produced. Specifically, for each subproblem, two candidate solutions are randomly selected among the subproblem’s neighbors (the closest weight vectors). Then, the proposed genetic crossover operator (explained in “The Proposed Genetic Operators”) is applied to them. Furthermore, a uniform mutation is used. The current candidate solution and all the neighbors are replaced with the newly obtained candidate solution if the new candidate solution performs better. This step is shown in lines 11–12 of Algorithm 1.

Step 3. Comparison and replacement: Because of the conflicting objectives in multi-objective optimization problems, the comparison of the candidate solutions is always challenging. To compare the new candidate solution with the available ones, we use a decomposition-based method. Specifically, the decomposition method tries to combine the objective functions and achieve a scalar function for making the comparison possible. Here, different combination methods can be used. The proposed method uses the Tchebycheff method to combine the objective functions. As mentioned in “Tchebycheff Method”, this method considers the distances between the objective values and the reference point. The distances of the first objective value (number of features) have larger values than the Hamming loss, and this affects the performance of the Tchebycheff method. Therefore, it is better to normalize the obtained values of the objective functions.

The Tchebycheff value for the new candidate solution is calculated using the weight vector of the current solution. Then, the new solution is compared with the current solution and any of its neighbors. The new solution replaces them if it is better than the previous solutions. Finally, we expect to achieve a population with an improved generation. The population contains the best solution for each subproblem found so far. Whenever a new solution is produced, the reference point also needs to be updated. These steps are shown in lines 13–31 of Algorithm 1.

Step 4. Local search and obtaining the Pareto front: To increase the efficiency of the proposed algorithm, we use a local search with the concept of crowding distance. The details of the local search are given in “Local Search”. The final set of nondominated solutions will be obtained from the archive. These steps are shown in lines 34–39 of Algorithm 1.

As it is mentioned before, a set of non-dominated solutions are obtained at the last part of the search process. These solutions are trade-off feature subsets with a variety of number of features and classification performance. A user has different options and accordingly can make a decision based on corresponding application. For example, if the user prefers less computational complexity, he/she can select one of the small subsets with a small value of sacrificing the classification accuracy. Multi-criteria decision making (MCDM) (Velasquez & Hester, 2013) is a process to rank and select from a set of candidate solutions with conflicting and non-commensurable criteria As an example, VIKOR (Bidgoli et al., 2019) is one of such methods which ranks the multi-criteria solutions based on the particular measure of closeness to the ideal point (a constructed point using minimum value of each objective).

## Experimental Design

### Datasets

For many applications, standard multi-label datasets are available. For each application, several datasets were selected to evaluate the proposed method. We consider the eight real-world multi-label datasets presented in Table 1. These are complex datasets with many labels and features. The feature numbers ranged between 19 and 1,449. Thus, feature selection in such datasets is considered as a large-scale optimization problem. Moreover, the number of labels is between 6 and 23. Two datasets were selected from the field of biology. The first one is the Yeast dataset (Elisseeff & Weston, 2002). In this dataset, genes can be placed in different activity groups. Genbase is another dataset in the field of biology, which is related to the protein families (Diplaris et al., 2005). There are 27 different groups of protein structures defined in this dataset. Every protein fiber can belong to one or several structures. In image classification, two datasets are used, Scene (Boutell et al., 2004) and Flags (Dheeru & Karra Taniskidou, 2017). The Scene dataset is related to the categories of desert, beach, mountain, etc. For example, a scene image can have the labels of “mountain” and “sea” at the same time. The Flags dataset contains information on flag images of different countries. In addition, we use multi-label text classification datasets to evaluate the efficiency of the proposed algorithm. The Medical dataset (Pestian et al., 2007) includes the classification of radiological reports in 45 different categories. The Enron (Klimt & Yang, 2004) dataset is another text classification dataset that includes the categories of the collected emails. In audio classification, the Emotions dataset includes the categories of feelings in music (Trohidis et al., 2008). The features of music have been extracted and categorized into 6 categories of feelings (surprised, amazed, happy, pleased, calm, relaxing). Each piece of music can include more than one feeling. The Birds dataset (Briggs et al., 2013) is related to the classification of birds using their recorded sounds. This dataset categorizes 19 species of birds.

**Table 1:** Multi-label datasets used in the experiments.

Datasets	Domain	#Training instances	#Test instances	#Labels	#Features
Emotions	Music	391	202	6	72
Scene	Image	1,211	1,196	6	294
Flags	Image	129	65	7	19
Yeast	Biology	1,500	917	14	103
Birds	Audio	322	323	19	260
Genbase	Biology	463	199	27	1,186
Medical	Text	645	333	45	1,449
Enron	Text	1,123	579	53	1,001

### Experimental setup

The multi-label dataset should be divided into training and test sets to evaluate the proposed method. During the optimization process, the performance of the selected features is evaluated by computing the accuracy of classification on training data. The test set is used to evaluate the obtained features and to report the final results at the end of the algorithm. The number of the training and test samples for each dataset is provided in Table 1. The splitting of data into test and train subsets is based on the Mulan library (Tsoumakas et al., 2011), which provided these datasets. The classifier algorithm used in multi-label learning is the ML-KNN classifier. To simplify the evaluation process, Euclidean distance with *k* = 10 was used in this study (Zhang & Zhou, 2007). Some parameters need to be adjusted before the implementation of the proposed algorithm. The population size and the number of the function evaluations used were 100 and 50,000, respectively, for all methods. The algorithms were run 40 times independently for each dataset. In the proposed method, the number of neighbors, *T*, was set to 10. The mutation rate in the genetic operators was 0.05. To increase the diversity of solutions, a random number between 0.1 and 0.9 was used as the value of parameter *F* in the local search based on Zhang et al. (2017). Finally, the values of parameters *P*_1_ and *P*_2_ for selecting the crossover operator were set to 0.1 and 0.2, respectively.

## Results and Discussion

Each dataset introduced in the previous section was given to the algorithm as the input data for evaluating the proposed method. Then, a set of nondominated solutions was reported at the end of the algorithm. For each dataset, the algorithms were run for 40 independent runs, and we obtained 40 Pareto fronts. The nondominated solutions in the union set of all Pareto fronts were determined as the best Pareto front. Therefore, all comparisons were carried out between the best Pareto fronts of the methods. The proposed method has been compared with the PSO-based algorithm (Zhang et al., 2017) and the NSGA-II-based (Yin, Tao & Xu, 2015) multi-label feature selection algorithm, which are explained in “Multi-label Feature Selection Using Multi-objective Evolutionary Algorithms” and the main version pf MOEA/D algorithm.

### Assessment metrics

A comparison of the multi-objective optimization methods in various applications is always challenging. Therefore, we should find assessment metrics that facilitate the comparison. In this study, we used an extensive set of measures to evaluate the efficiency of the proposed algorithm.

Some assessment measures focus on one of the objective functions. The minimum number of the obtained features and the minimum classification error on the training and test sets are single-objective criteria for feature selection. Because there may be some subsets with only a few features but a large classification error, we consider the subsets with a lower classification error instead of those using all features.

**Figure 6:**
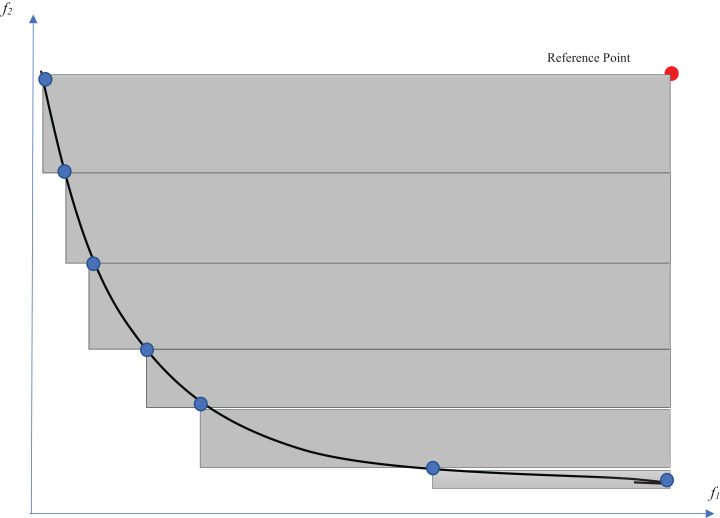
Hypervolume indicator.

From the multi-objective point of view, there are many evaluation measures that compare the obtained Pareto fronts of competitors (Akkan & Gülcü, 2018). The hypervolume indicator (Auger et al., 2009) is one of the well-known criteria for evaluating multi-objective optimization methods. This indicator evaluates multi-objective optimization algorithms according to both diversity and convergence to the optimal Pareto front. This indicator determines the volume of the *n*-dimensional space that is surrounded by a set of points. The number of dimensions would be equal to the number of objectives. Therefore, the volume of the two-dimensional space that is surrounded by the Pareto solutions is calculated for the feature selection problem. The larger this space, the wider the points (surrounding a larger space) and the closer the Pareto front to the optimal Pareto. A reference point is needed to acquire the intended volume. The selection of a reference point is one of the challenges for the calculation of the hypervolume indicator. For example, a point with the worst obtained values among the objective functions is an option for this purpose. As shown in Fig. 6, the volume of the gray regions between the solutions on the Pareto front and the reference point would be considered as the hypervolume indicator. The measure is defined in Eq. (14) (Auger et al., 2009):

(14) $${HV(A) = } {{\rm vol}\;\left(\bigcup\limits_{a \in A} [{f_1}(a),{r_1}] \times [{f_2}(a),{r_2}] \times \ldots \times [{f_M}(a),{r_M}]\right),}$$

where *a* ∈ *A* is a point at which all candidate solutions are weakly dominated by it.

The coverage of two sets is another metric for comparing the Pareto front obtained by multi-objective methods (Zitzler & Thiele, 1998). This criterion uses the domination concept. Coverage (A, B) for two algorithms A and B is the total number of obtained solutions on the final Pareto front of algorithm A that dominates the solutions of the Pareto front of algorithm B. In this way, coverage (B, A) indicates the number of obtained solutions of B that dominate the Pareto points of A. To evaluate the statistical significance of the obtained results, we conducted the Friedman test (Calzada-Ledesma et al., 2018) with a confidence interval of 95%. The ranks computed by means of the non-parametric test are reported for the obtained results. In each table, the last row indicates the statistical test results. *w/t/l* represents the number of wins, ties, and losses of the proposed method comparing to other algorithms.

### Results and analysis

In this section, we report the results of the comparison between the proposed method and two other multi-objective methods for multi-label data feature selection. Table 2 shows the minimum Hamming loss on the training and test data in different datasets. The second column indicates the classification error on the test data using all features without feature selection. The other columns show the classification error on training and test data by applying feature selection methods. Reducing the Hamming loss of classification using feature selection is evidence of existing irrelevant features in all datasets. Table 2 shows that the proposed method has achieved significantly better results compared with the other two methods on the training set. It obtained a smaller Hamming loss in 6 a out of 8 in comparison with the PSO method. The results are even better comparing to MOEA/D and NSGA-II, so that it has reached less error on 7 out of 8 datasets. Regarding the test data, the proposed algorithm shows a better performance in most of the datasets. It outperforms the PSO and MOEA/D in 6 out of 8 datasts. Comparing to NSGA-II, The proposed method has better accuracy on 7 out of 8 datasets.

**Table 2:** Comparison on minimum Hamming loss (HL) on training and test sets. The second column represents the HL values of the classification using all features. Highlighted numbers in the table indicate which method reaches lower Hamming Loss on each dataset.

Datasets	HL using all features	Minimum training HL using feature selection				Minimum testing HL using feature selection			
		Proposed method	PSO-based method	NSGA-II method	MOEA/D method	Proposed method	PSO-based method	NSGA-II method	MOEA/D method
Emotions	0.2137	**0.1343**	0.1415	0.1462	0.1368	**0.1939**	0.2030	0.2013	0.1955
Scene	0.0962	**0.0587**	0.0635	0.0637	0.0601	**0.0909**	0.0943	0.0930	0.0916
Yeast	0.2005	**0.1636**	0.1666	0.1690	0.1665	**0.1962**	0.1979	0.1995	0.1986
Birds	0.0481	**0.0371**	0.0404	0.0407	0.386	**0.0442**	0.0445	0.0460	**0.0442**
Genbase	0.0043	**0.0015**	**0.0015**	0.0020	0.0022	**0.0023**	**0.0022**	0.0030	**0.0022**
Medical	0.0153	**0.0080**	0.0086	0.0096	0.0083	**0.0105**	0.0106	0.0111	0.0107
Enron	0.052	**0.0399**	0.0421	0.0428	0.0443	0.0495	**0.0490**	0.0498	0.0501
Flags	0.3099	**0.2004**	**0.2004**	**0.2004**	**0.2004**	0.2615	0.2527	**0.2396**	0.2549
w/t/l		–	**6/2/0**	**7/1/0**	**7/1/0**	–	**6/1/1**	**7/0/1**	**6/2/0**

If the methods are evaluated in terms of the number of features in the obtained subsets, regardless of the classification error, the solutions that have few features and high classification errors would be reported as desirable solutions. Therefore, to give a reasonable comparison, solutions that achieved a lower classification error than using all features are selected. The minimum number of the selected features among these solutions is reported in Table 3. In fact, this table represents the smallest subset of features which could obtain a classification with Hamming loss less than using all features. Therefore the minimum number of test features in this table indicates that which subset of features with minimum number of features could reach less error than all features on test subset. In most cases, the proposed method selected a lower number of features than two other methods. The difference between the number of selected features using the proposed method and competitors is remarkable; e.g., the proposed method has achieved a smaller Hamming loss than other methods on the test set of the Birds dataset using only 5 features, whereas this number is 10 in the PSO-based method and 90 in NSGA-II, which means that the proposed method has further reduced the number of features by one-fiftieth. In the Genbase dataset, the proposed method has decreased the number of features to 52, while the main dataset has 1,186 features.

**Table 3:** Comparison on smallest feature subset with Hamming loss less than all features obtained by each method on training and test sets. Highlighted numbers in the table indicate which method reaches smaller feature subsets on each dataset.

Datasets	All features	Minimum number of training features				Minimum number of test features			
		Proposed method	PSO-based method	NSGA-II method	MOEA/D method	Proposed method	PSO-based method	NSGA-II method	MOEA/D method
Emotions	72	**2**	**2**	14	3	14	**8**	20	22
Scene	294	**15**	**15**	92	48	**104**	141	111	85
Yeast	103	**3**	4	19	8	41	**21**	54	50
Birds	260	**7**	9	80	39	**5**	10	90	47
Genbase	1,185	**73**	124	528	371	**52**	117	513	389
Medical	1,449	**121**	178	616	468	**146**	178	617	490
Enron	1,001	**18**	43	404	286	**41**	64	410	299
Flags	19	**1**	**1**	**1**	**1**	**1**	**1**	**1**	**1**
w/t/l		–	**5/3/0**	**7/1/0**	**7/1/0**	–	**5/1/2**	**7/1/0**	**7/1/0**

#### Results based on set-coverage metric

As mentioned in the previous section, the set coverage criterion is another measure used to compare the proposed method with other multi-objective feature selection methods. Table 4 shows the values of set coverage obtained by the different methods. In this table, ‘A’ indicates the proposed method, and ‘B’ is the competitor. Therefore, C (A, B) shows the average ratio of the number of solutions obtained by the proposed algorithm in each run that dominate the solutions of another algorithm. The mean, median, and standard deviation of 2-set coverage values are reported on training and test data in Table 4. As shown in the table, the proposed method achieved comparable results to the PSO-based method on the training set but a significant improvement on the test set. The results show that in the datasets with large numbers of features, such as Genbase, Medical, and Enron, the proposed method achieved significantly better results than the others. For example, on the training results in the Genbase dataset, 65% of solutions of the proposed method dominate PSO-based solutions, while this number is just 10% for PSO-based solutions. Therefore, the proposed method showed a high efficiency compared to two other algorithms for large-scale optimization problems. In comparing to NSGA-II and MOEA/D, the proposed method achieved significantly better results on all datasets. It means that the obtained Pareto front completely dominates obtained solutions by these algorithms.

**Table 4:** Comparison on 2-set coverage indicator of training and test data. C (A, B) is the value of the 2-set coverage between the proposed method and competitor. Highlighted numbers in the table indicate which method reaches higher coverage on each dataset.

Datasets		Training data						Test data					
		PSO-based method (B)		NSGA-II method (B)		MOEA/D method (B)		PSO-based method (B)		NSGA-II method (B)		MOEA/D method (B)	
		C (A, B)	C (B, A)	C (A, B)	C (B, A)	C (A, B)	C (B, A)	C (A, B)	C (B, A)	C (A, B)	C (B, A)	C (A, B)	C (B, A)
Emotions	Mean	0.266	**0.548**	**1.000**	0.000	**0.750**	0.050	0.635	**0.646**	**0.918**	0.192	**0.888**	0.000
	Median	0.270	0.533	1.000	0.000	0.703	0.010	0.714	0.657	1.000	0.200	0.745	0.000
	Std	0.111	0.103	0.000	0.000	0.103	0.110	0.245	0.149	0.121	0.080	0.216	0.000
Scene	Mean	0.283	**0.373**	**1.000**	0.000	**1.000**	0.000	**0.551**	0.343	**0.847**	0.035	**0.470**	0.322
	Median	0.263	0.425	1.000	0.000	1.000	0.000	0.461	0.375	0.750	0.013	0.495	0.319
	Std	0.156	0.088	0.000	0.000	0.000	0.000	0.212	0.111	0.141	0.041	0.173	0.103
Yeast	Mean	0.320	**0.467**	**0.995**	0.000	**0.636**	0.272	**0.680**	0.555	**0.949**	0.174	**0.875**	0.066
	Median	0.295	0.483	1.000	0.000	0.620	0.301	0.659	0.580	1.000	0.171	0.837	0.060
	Std	0.144	0.090	0.027	0.000	0.030	0.013	0.119	0.181	0.065	0.093	0.240	0.172
Birds	Mean	**0.453**	0.221	**1.000**	0.000	**1.000**	0.000	**0.534**	0.429	**0.969**	0.039	**1.000**	0.000
	Median	0.455	0.211	1.000	0.000	1.000	0.000	0.536	0.474	1.000	0.000	1.000	0.000
	Std	0.238	0.123	0.000	0.000	0.000	0.000	0.311	0.144	0.096	0.059	0.000	0.000
Genbase	Mean	**0.651**	0.108	**1.000**	0.000	**0.714**	0.000	**0.606**	0.167	**0.996**	0.000	**1.000**	0.000
	Median	0.845	0.091	1.000	0.000	0.719	0.000	0.768	0.134	1.000	0.000	1.000	0.000
	Std	0.391	0.094	0.000	0.000	0.140	0.000	0.364	0.131	0.022	0.000	0.000	0.000
Medical	Mean	**0.360**	0.202	**1.000**	0.000	**1.000**	0.000	**0.579**	0.287	**1.000**	0.001	**1.000**	0.000
	Median	0.333	0.205	1.000	0.000	1.000	0.000	0.638	0.302	1.000	0.000	1.000	0.000
	Std	0.267	0.102	0.000	0.000	0.000	0.000	0.267	0.095	0.000	0.005	0.000	0.000
Enron	Mean	**0.331**	0.298	**1.000**	0.000	**1.000**	0.000	**0.746**	0.602	**0.942**	0.281	**1.000**	0.000
	Median	0.325	0.302	1.000	0.000	1.000	0.000	0.793	0.628	1.000	0.279	1.000	0.000
	Std	0.190	0.069	0.000	0.000	0.000	0.000	0.224	0.042	0.120	0.032	0.000	0.000
Flags	Mean	0.014	**0.544**	**0.912**	0.057	**0.166**	0.000	0.447	**0.579**	**0.865**	0.298	0.142	**0.400**
	Median	0.000	0.519	1.000	0.000	0.166	0.000	0.464	0.600	1.000	0.286	0.137	0.350
	Std	0.057	0.105	0.170	0.135	0.000	0.000	0.103	0.115	0.202	0.192	0.249	0.130
w/t/l		–	**4/0/4**	–	**8/0/0**	–	**8/0/0**	–	**6/0/2**	–	**8/0/0**	–	**7/0/1**

Similar results are repeated for the test data, whose results are more important than the training data. For test data, the proposed algorithm had a higher 2-set coverage value than the PSO-based algorithm in all datasets except Flags and Emotions. The difference in most datasets is significant. On datasets like Genbase and Medical, 2-set coverage of the proposed method is two times more than competitors. For example, in the Genbase dataset, the proposed method dominates 60% of PSO-based solutions, while the PSO-based method dominates only 16% of the others’ solutions. Regarding the NSGA-II method, the value of the 2-set coverage of the proposed method is significantly higher than the NSGA-II method on all datasets. The proposed method even reached 100% 2-set coverage in the Medical dataset. It means that all solutions of the proposed method dominated the solutions obtained by the NSGA-II algorithm at each run. Also this table shows the compared results of 2-set coverage on MOEA/D and the proposed method. On most of the datasets, proposed method dominates all obtained solutions using MOEA/D algorithm. It confirms that the modification of the algorithm, specially adding the local search, leads finding more non-dominated solutions.

#### Results based on hypervolume metric

The hypervolume indicator is another multi-objective measure that compares the results of the proposed method with other algorithms. The value of this criterion was calculated for the Pareto front obtained in each run. The comparison of the mean, median, and standard deviation of hypervolume indicators for 40 independent runs on each dataset is provided in Table 5. In most of the datasets, the hypervolume indicator of the proposed method is more than 0.9, while for two other methods, all the values are less than 0.9. For NSGA-II, the hypervolume values are between 0.5 and 0.6. The PSO-based and MOEA/D methods outperform the proposed method in terms of the hypervolume indicator only on the Flags dataset. Otherwise, the proposed method had significantly better results compared to other algorithms. Therefore, as this measure indicates, the proposed method presents well-distributed solutions that are closer to the optimal Pareto front. The ranks computed by means of non-parametric Friedman tests are reported at the end of Table 6 based on 2-set coverage and hypervolume metric. Notice that the smallest rank corresponds to the best-performing method.

**Table 5:** Comparison on hypervolume indicator of training and test Pareto fronts. Highlighted numbers in the table indicate which method reaches higher hypervolume indicator on each dataset.

Datasets		Hypervolume of training Pareto front				Hypervolume of test Pareto front			
		Proposed method	PSO-based method	NSGA-II method	MOEA/D method	Proposed method	PSO-based method	NSGA-II method	MOEA/D method
Emotions	Mean	**0.849**	0.820	0.647	0.828	**0.776**	0.750	0.607	0.770
	Median	0.849	0.820	0.647	0.828	0.774	0.750	0.607	0.770
	Std	0.000	0.000	0.000	0.000	0.005	0.001	0.000	0.000
Scene	Mean	**0.932**	0.780	0.324	0.783	**0.898**	0.752	0.313	0.758
	Median	0.932	0.863	0.591	0.783	0.898	0.831	0.570	0.758
	Std	0.000	0.019	0.021	0.001	0.018	0.021	0.000	0.000
Yeast	Mean	**0.827**	0.794	0.570	0.780	**0.796**	0.763	0.550	0.751
	Median	0.827	0.794	0.570	0.780	0.796	0.763	0.550	0.751
	Std	0.000	0.000	0.000	0.001	0.000	0.000	0.000	0.000
Birds	Mean	**0.959**	0.920	0.630	0.830	**0.953**	0.915	0.627	0.826
	Median	0.958	0.920	0.630	0.830	0.952	0.915	0.627	0.826
	Std	0.001	0.000	0.000	0.000	0.001	0.000	0.000	0.000
Genbase	Mean	**0.995**	0.854	0.568	0.990	**0.996**	0.854	0.569	0.688
	Median	0.995	0.854	0.568	0.990	0.995	0.854	0.569	0.688
	Std	0.001	0.000	0.000	0.000	0.001	0.000	0.000	0.000
Medical	Mean	**0.989**	0.833	0.552	0.672	**0.987**	0.831	0.551	0.670
	Median	0.989	0.833	0.552	0.672	0.987	0.831	0.551	0.670
	Std	0.000	0.000	0.000	0.000	0.000	0.000	0.000	0.000
Enron	Mean	**0.957**	0.819	0.492	0.682	**0.949**	0.813	0.489	0.678
	Median	0.957	0.819	0.492	0.682	0.949	0.813	0.489	0.678
	Std	0.000	0.000	0.000	0.000	0.000	0.000	0.000	0.000
Flags	Mean	0.781	**0.786**	0.736	**0.785**	0.731	0.731	0.687	**0.737**
	Median	0.781	0.786	0.736	0.785	0.731	0.731	0.687	0.737
	Std	0.001	0.001	0.000	0.000	0.000	0.000	0.000	0.000
w/t/l		–	**7/0/1**	**8/0/0**	**7/0/1**	–	**7/1/0**	**8/0/0**	**7/0/1**

**Table 6:** Ranks of feature selection methods according to Friedman test. Highlighted numbers in the table indicate which method reaches best rank.

	Proposed method	PSO-based	NSGA-II	MOEA/D
2-Set Coverage	**1.034**	2.14	2.85	2.57
Hypervolume indicator	**1.012**	2.26	2.73	2.52

#### Results based on Pareto fronts

**Figure 7:**
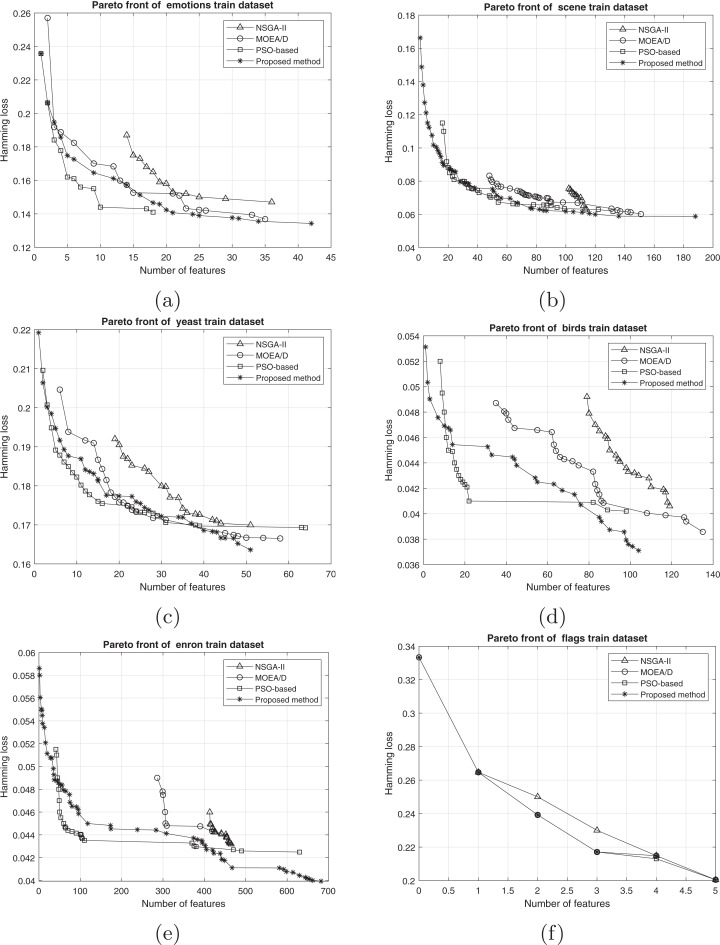
Train Pareto front on training data including (A) emotions, (B) scene, (C) yeast, (D) birds, (E) enron, and (F) flags.

**Figure 8:**
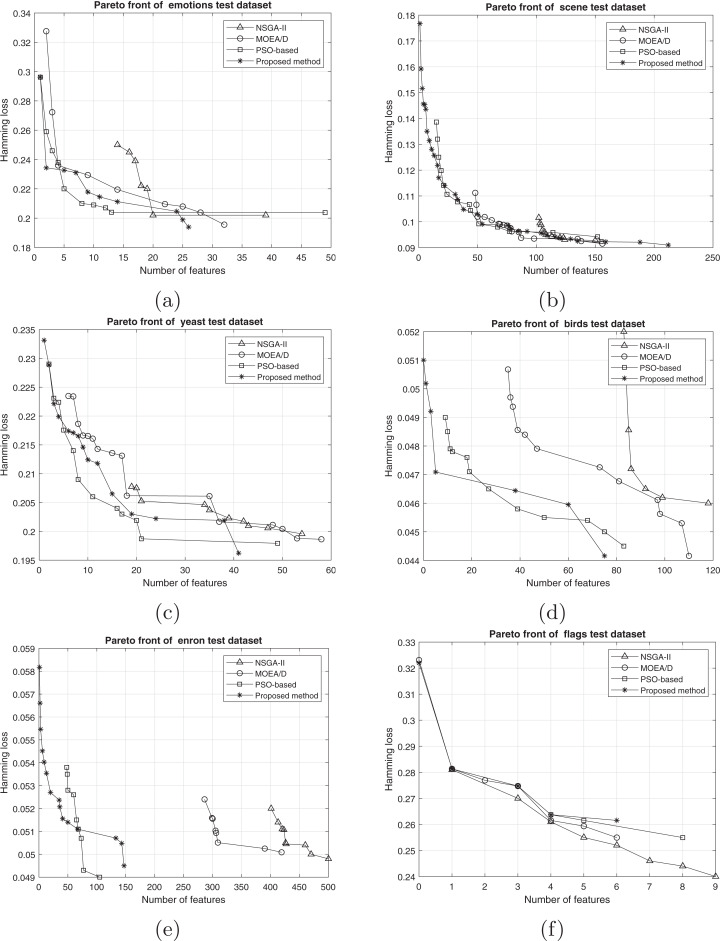
Pareto front on test data including (A) emotions, (B) scene, (C) yeast, (D) birds, (E) enron, and (F) flags.

The comparison between the best obtained Pareto fronts in different methods on training and test sets is shown in Figs. 7 and 8. The proposed method has a better Pareto front in most of the datasets compared to the other methods in terms of both the number of features and the classification performance. The diversity of the obtained solutions is one of the properties of the obtained Pareto front of the proposed method. In datasets such as Genbase, the proposed method has a better Pareto front than other methods. It means that, with equal numbers of features, the proposed method achieved a lower Hamming loss. In some datasets such as Birds, Emotions, and Enron, the Pareto front obtained from the PSO-based method has a better performance than the proposed method only in a few regions (mostly in the middle part of the Pareto front). However, we can see that the 2-set coverage of the proposed method is higher in most of the datasets, according to Pareto fonts. Based on a comparison of the hypervolume indicator, it is obvious that wider Pareto fronts lead to higher values of hypervolume on all datasets. Furthermore, the proposed algorithm has a considerably better performance than the NSGA-II algorithm in all datasets, and as discussed previously, all the solutions of NSGA-II are dominated using the proposed method. NSGA-II has a smaller Pareto front compared to other methods. This algorithm could find just a small number of solutions in the search space. Regarding the comparison between the proposed method and the original version of MOEA/D, as it is presented in the plots, the proposed method obtains the wider Pareto front with more non-dominated solutions. On most of the datasets, MOEA/D Pareto front is placed between NSGA-II and PSO PFs (i.e., better than NSGA-II but worse than PSO) while the proposed method dominates most of the obtained solutions of those methods. It means that modification on original algorithm has improved the performance of process of the multi-objective search. The local search strategy finds better solutions in terms of dominance, however, MOEA/D algorithm doesn’t utilize dominance concept to select the best solutions. Therefor, as it is obvious, a combination of decomposition and dominance can find more promising regions in the search space. This strategy improves the exploitation power of the algorithm. On the other hand, applying a combination of crossover operators increases the exploration power of the search algorithm. Another advantage of the obtained Pareto front in the proposed algorithm is the ability of the method to search in the scope of the solutions with a low number of features while the other two methods have not obtained such solutions. This issue is more prominent regarding the NSGA-II algorithm, and the solutions obtained by this method have not been as successful as the other two methods in decreasing the number of features. The number of features in the proposed method is usually smaller than PSO-based and NSGA-II obtained features. As it is concluded from experiments, the proposed method can find a set of feature subsets which in multi-label classification tasks can be used. In each multi-label classification such as all applications which are experimented in this paper, we need to select the best features. Therefore, based on MCDM, a set of features can be selected among subsets on the Pareto front to improve the performance of the classification.

## Concluding remarks

Many real-world applications require multi-label classification. If more than one label exists, feature selection plays a significant role in the performance of the multi-label classification. The main purpose of this paper is to propose a decomposition-based evolutionary multi-objective method for selecting optimal features in multi-label data. The multi-objective search space is divided into several scalar subproblems so that each subproblem can be solved using evolutionary algorithms. It yields a more effective search process to improve the quality of the obtained solutions. To increase the efficiency of the proposed method, a local search is used. This gives more exploitation power for finding better feature subsets. In addition, a combination of the various crossover operators results in more diverse solutions. The results indicate that this method achieves a better Pareto front in most of the standard multi-label datasets compared to the other methods in terms of both the number of features and the classification performance. Furthermore, the progress of the results in other evaluation criteria, such as the 2-set coverage and hypervolume indicator, represents a considerable performance improvement of the proposed method compared to the previous methods. Despite the satisfactory performance of the proposed method, several points are considered for future investigations. The efficiency of the proposed algorithm can be examined in more problems, especially in more real-world applications. In the present study, only two objectives were evaluated. Additional objective functions may be considered in future work. Moreover, the proposed method uses continuous genetic operators, which can be replaced with binary operators to improve the performance.

## Supplemental Information

10.7717/peerj-cs.261/supp-1 Supplemental Information 1 Multi-label classification datasets.

10.7717/peerj-cs.261/supp-2 Supplemental Information 2 MATLAB Code. Code is written in MATLAB language. It starts with Demo.m file. The path of the data should be determined in this file.

## Additional Information and Declarations

### Data Availability

The following information was supplied regarding data availability: Data and code are available in the Supplemental Files. Our experimental data are multi-label classification datasets taken from Mulan library: http://mulan.sourceforge.net/datasets-mlc.html.
